# Design of Pd–Zn Bimetal MOF Nanosheets and MOF-Derived Pd_3.9_Zn_6.1_/CNS Catalyst for Selective Hydrogenation of Acetylene under Simulated Front-End Conditions

**DOI:** 10.3390/molecules27175736

**Published:** 2022-09-05

**Authors:** Xinxiang Cao, Ruijian Tong, Siye Tang, Ben W. -L. Jang, Arash Mirjalili, Jiayi Li, Xining Guo, Jingyi Zhang, Jiaxue Hu, Xin Meng

**Affiliations:** 1 Laboratory for Development & Application of Cold Plasma Technology, College of Chemistry and Chemical Engineering, Luoyang Normal University, Luoyang 471022, China; 2 School of Agriculture and Bioengineering, Heze University, Heze 274015, China; 3 Department of Chemistry, Texas A&M University-Commerce, Commerce, TX 75429-3011, USA; 4 Department of Chemistry, University of California, Riverside, CA 92521, USA

**Keywords:** metal–organic framework, porphyrin, nanosheet, palladium–zinc intermetallic, carbonization, selective hydrogenation, acetylene

## Abstract

Novel zinc–palladium–porphyrin bimetal metal–organic framework (MOF) nanosheets were directly synthesized by coordination chelation between Zn(II) and Pd(II) tetra(4-carboxyphenyl)porphin (TCPP(Pd)) using a solvothermal method. Furthermore, a serial of carbon nanosheets supported Pd–Zn intermetallics (Pd–Zn-ins/CNS) with different Pd: Zn atomic ratios were obtained by one-step carbonization under different temperature using the prepared Zn-TCPP(Pd) MOF nanosheets as precursor. In the carbonization process, Pd–Zn-ins went through the transformation from PdZn (650 °C) to Pd_3.9_Zn_6.1_ (~950 °C) then to Pd_3.9_Zn_6.1_/Pd (1000 °C) with the temperature increasing. The synthesized Pd–Zn-ins/CNS were further employed as catalysts for selective hydrogenation of acetylene. Pd_3.9_Zn_6.1_ showed the best catalytic performance compared with other Pd–Zn intermetallic forms.

## 1. Introduction

Since the discovery of graphene in 2004, two-dimensional (2D) nanomaterials have attracted much research interest due to their unique electronic structure and fascinating physical and chemical properties [1,2]. Up to now, a variety of 2D nanomaterials, such as graphene oxide, carbon nitride, boron nitride, black phosphorus, metal oxide, metal hydroxides and transition metal chalcogenides, etc., have been reported [1,3]. However, those 2D nanomaterials are mostly inorganic compounds, and they are simple in composition and lack diversity in structure. Metal–organic framework (MOF) nanosheets are a new kind of 2D nanomaterial that has received increasing attention recently, owing to their high porosity, large surface area, easy access to active sites and numerous structural possibilities [1,3,4,5]. The use of 2D MOF nanosheets is showing potential for application in many fields, such as molecular sieving [6,7], luminescent sensing [8,9], energy storage and conversion [10,11], catalysis [12,13] and biomedicine [14,15]. Conventionally, the preparation of 2D MOF nanosheets usally requires special preparation strategies, such as ultrasonication exfoliation [16] layer-by-layer growth, [17], three-layer synthesis [18], liquid exfoliation [1] and surfactant-assisted synthesis [19]. However, the direct synthesis of 2D MOF nanosheets is still rare, particularly, the direct synthesis of 2D bimetal MOF nanosheets has not been reported yet [20,21,22,23]. As far as we know, only the 2D 1,4-benzene dicarboxylate (BDC)-based (CuBDC [24] and ZnBDC [25]) and tetrakis(4-carboxyphenyl)porphyrin (TCPP)-based (ZnTCPP [26] and Gd-TCPP [3]) MOF nanosheets have been reported.

Removing trace amount of acetylene (~1%) from ethylene feed is commercially important because acetylene will poison Ziegler–Natta ethylene-polymerization catalysts [27]. Selective hydrogenation is the most effective and widespread method in industry for diminishing the acetylene impurity to an acceptable level and re-utilizing it as the raw material for the polyethylene process [28]. Palladium is the most active metal for selective hydrogenation of acetylene, as a result, palladium-based catalysts are the most widely used in industry for this reaction. On the other hand, palladium nanoparticles also have an inherent disadvantage for this reaction, i.e., poor selectivity to ethylene [29]. To overcome this, a great effort has been made by researchers, including adding a second metal, such as Au, Ag, Ga, Zn, Sn, In, Pb, Co or Cu, to modify the nature of Pd, and the use of organic N, S or P as modifiers to partially cover the Pd active sites, etc., [30,31]. Despite the significant research progress achieved by Pd-based catalysts for selective hydrogenation of acetylene, a simultaneous optimization of conversion and selectivity is still very challenging, especially in the front-end conditions where H_2_ is in large excess [32]. Recently, it was found that supported palladium–zinc alloy catalysts exhibited high performance for the hydrogenation of acetylene in the front-end hydrogenation process [33,34,35,36,37]. According to the literature, the type of Pd–Zn intermetallics (Pd–Zn-ins) in catalysts dramatically affected the activity and selectivity of specific catalytic reactions [38,39,40,41]. However, to the best of our knowledge, the synthesis of a serial of Pd–Zn-ins and the discussion of the effect of the intermetallic type on selective hydrogenation of acetylene have not been reported. 

Herein, we developed a novel porphyrin-based palladium–zinc-bimetal–organic framework (Zn–TCPP(Pd)) nanosheets. Subsequently, the resulting MOF was further thermally carbonized at different temperatures under an inert atmosphere to obtain a series of carbon nanosheet supported Pd–Zn intermetallics (Pd–Zn-ins/CNS) with different Pd: Zn atomic ratios. The elemental composition, microstructure and morphology of the obtained MOF and Pd–Zn-ins/CNS were comprehensively characterized by FT-IR, XRD, SEM, TEM, BET and ICP. Moreover, the transition trend of Pd–Zn intermetallic with increasing temperature was investigated. Furthermore, the catalytic performance of the Pd–Zn-ins/CNS for selective hydrogenation of acetylene in the simulated front-end hydrogenation process condition was tested, and the effect of the Pd–Zn intermetallic type in the catalysts on the reaction was discussed. 

## 2. Results and Discussion

**Figure 1:**
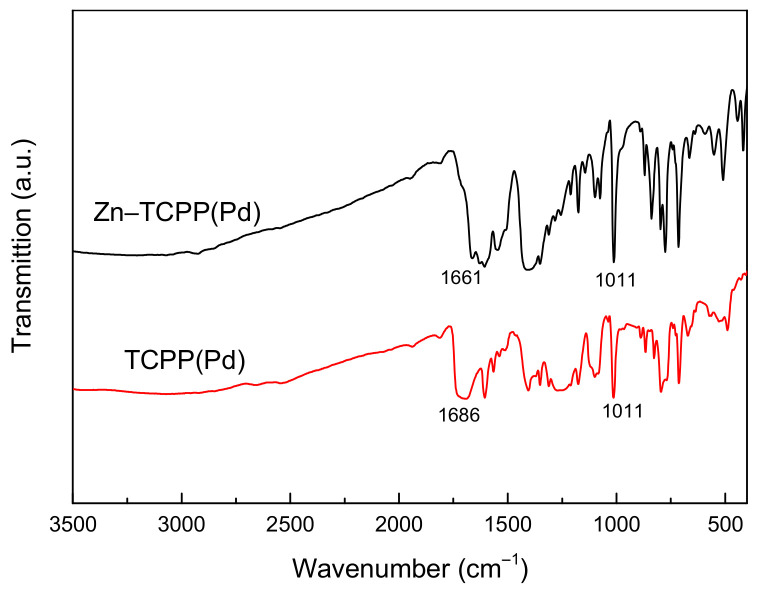
FT-IR spectra of TCPP(Pd) and the prepared Zn–TCPP(Pd) MOF nanosheets.

The Zn–TCPP(Pd) MOF nanosheets were fabricated via a simple solvothermal method only using Zn(II) nitrate hexahydrate and Pd (II) tetra(4-carboxyphenyl) porphin (TCPP(Pd)) in the mixed solvent of DMF and ethanol, by coordination chelation between Zn ions and the carboxyl functional groups. The formation of Zn–TCPP(Pd) MOF was investigated by Fourier transform infrared spectroscopy (FT-IR), as shown in Figure 1. The absorption peaks observed at 1011 cm^−1^ in the both the spectra of TCPP(Pd) and Zn–TCPP(Pd) are assigned to the Pd–N bond, indicating the presence of Pd^2+^ ions in the porphyrin ring of TCPP. This is well in line with the results of other studies [42]. Moreover, the absorption peaks at 1683 cm^−1^ in the spectrum of TCPP(Pd) are due to the C = O stretching vibration from the carboxyl functional groups [3], however, this is significantly weakened and a new absorption peak appears at 1661 cm^−1^ in the spectrum of Zn–TCPP(Pd). These phenomena were also observed and thought to result from the formation of coordination bond between metal ions and COOH by Zhao et al. [3]. That is, Zn ions were successfully coordinated with COOH in TCPP(Pd) to form a new MOF, Zn–TCPP(Pd) in our case. Inductively coupled plasma mass spectrometry (ICP-MS) showed that the molar ratio of Pd/Zn in the prepared Zn–TCPP(Pd) MOF is approximately 1:2 (Table 1).

**Figure 2:**
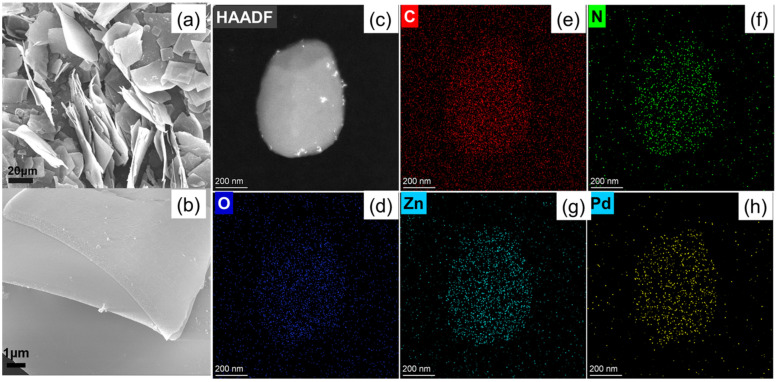
(**a**) SEM, HAADF-TEM (**b**) and TEM-EDS (**c–h**) mapping images of the prepared Zn–TCPP(Pd) MOF nanosheets.

Moreover, the morphology and chemical composition of the prepared Zn–TCPP(Pd) MOF nanosheets were characterized by field-emission scanning electron microscopy (SEM), high-angle annular dark-field transmission electron microscopy (HAADF-TEM) and energy dispersive X-ray spectra (EDS). As shown in Figure 2a,b, the prepared Zn–TCPP(Pd) are all sheets of irregular morphology and with the sizes of tens of microns. The thickness of the sheets measured by software (Nano Measurer) is only about tens of nanometers. According to the HAADF-TEM image and EDS mapping images as shown in Figure 2c–h, it can be confirmed that the prepared MOF nanosheets contained O, C, N, Zn and Pd elements, which are dispersed uniformly. 

**Figure 3:**
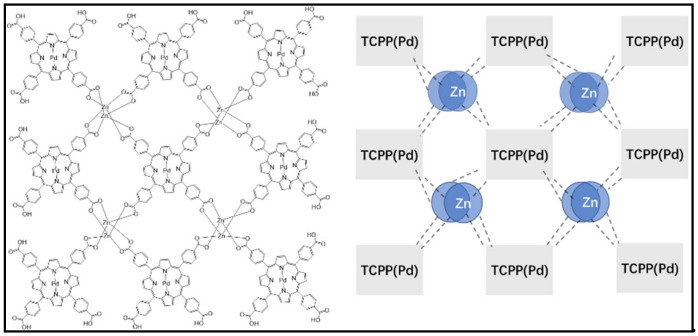
The proposed molecular structure of the prepared Zn–TCPP(Pd) MOF nanosheets.

Zhao et al. [26] reported a novel Zn–TCPP(Zn) MOF. The experimental and theoretical calculation results showed that the molar ratio of Zn to TCPP in Zn–TCPP(Zn) MOF was 3:1. Each porphyrin ring immobilizes a zinc ion that was coordinated with all four N atoms of the ring; for the remaining zinc ions, two zinc ions coordinated with four nearby carboxyl groups belonging to four different TCPP forming the two-dimensional topology of Zn–TCPP(Zn). Based on the above FT-IR and SEM/TEM-EDS characterization and ICP-MS analysis results as well as the previously reported molecular structure of Zn–TCPP(Zn) MOF [26], a logical molecular structure of the prepared Zn–TCPP(Pd) MOF nanosheets is proposed in Figure 3. Each zinc should be tetracoordinated, and is coordinated with two hydroxyl oxygens and two carbonyl oxygens of the four carboxyl groups, from four TCPP(Pd), respectively, forming a two-dimensional topology.

**Figure 4:**
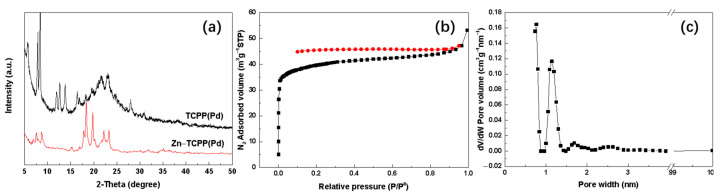
(**a**) XRD patterns of TCPP(Pd) and the prepared Zn–TCPP(Pd) MOF nanosheets, (**b**) N_2_ adsorption–desorption isotherms and (**c**) pore size distribution of the prepared Zn–TCPP(Pd) MOF nanosheets.

Additionally, X-ray powder diffraction (XRD) spectroscopy analysis for TCPP(Pd) and the prepared Zn–TCPP(Pd) MOF nanosheets as well as the N_2_ adsorption/desorption tests for the Zn–TCPP(Pd) MOF were performed in this study. As shown in Figure 4a, the XRD peaks of TCPP(Pd) before and after coordination with zinc ions obviously changed in (2θ) position and intensity, indicating the formation of new a crystal substance due to the coordination of Zn ions and TCPP(Pd). The XRD pattern of TCPP(Pd) coincides with the pattern reported by Cao et al. [43]. However, for the Zn–TCPP(Pd) MOF nanosheets, although the XRD pattern is basically consistent with those in literature [44], there are also obvious differences. This is probably due to the fact that the reported Zn–TCPP(Pd) MOFs are either bulk crystals rather than nanosheets, or intercalated crystals with different pillaring struts [44].

The as-prepared MOF nanosheets displayed an approximate type I Langmuir isotherms with a Brunauer–Emmett–Teller surface area (S_BET_) of 125.6 m^2^ g^−1^, average pore size of 2.3 nm, and pore volume of 0.08 cm^3^ g^−1^ (Figure 4b and Table 1). It is worth noting that the pores are basically classified as micropores, and the pore size distribution is very uniform with mainly 0.8 nm and 1.2 nm pores (Figure 4c). The relatively high S_BET_ and uniformly microporous structure may allow potential applications of the prepared Zn–TCPP(Pd) MOF nanosheets in many areas.

**Figure 5:**
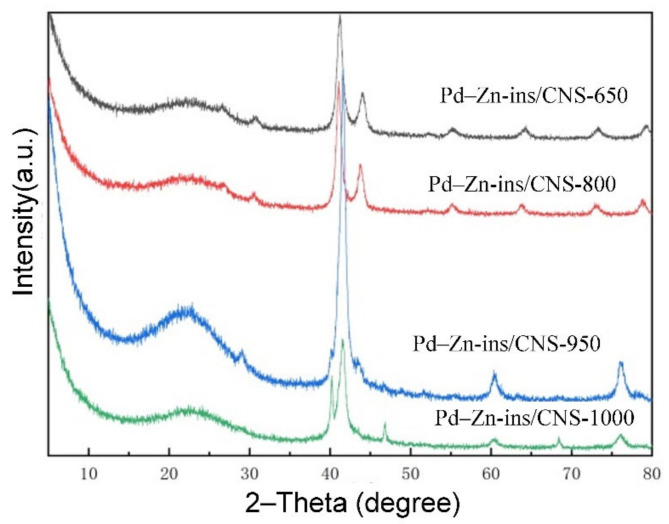
XRD patterns of Pd–Zn-ins/CNS-650, Pd–Zn-ins/CNS-800, Pd–Zn-ins/CNS-950, and Pd–Zn-ins/CNS-1000.

Further, the prepared Zn–TCPP(Pd) MOF nanosheets were carbonized in N_2_ at 650 °C, 800 °C, 950 °C and 1000 °C for 3 h, and the crystal phase composition and the microstructures of the resulting samples were analyzed. As shown in Figure 5, there is a broad peak at 2θ = 20°−25°, in all XRD patterns that is attributed to the characteristic diffraction peak of amorphous carbon [45]. For the patterns of samples were carbonized at 650 and 800°C, i.e., Pd–Zn-ins/CNS-650, Pd–Zn-ins/CNS-800, the diffraction peaks, located at 2θ = 26.8°, 30.8°, 41.2°, 44.1°, 55.2°, 64.2°, 72.9 and 79.2° correspond to the lattice planes of (001), (110), (111), (200), (002), (112), (310) and (311) of PdZn, respectively [34,35,36,37,38,39,40,41]. No diffraction peaks of individual palladium and zinc or their oxides were detected. When the carbonization temperature was raised to 950 °C, all the PdZn diffraction peaks almost disappear and new obvious peaks appear at 2θ = 29.6°, 42.0°, 60.9° and 76.7° that are attributed to Pd_3.9_Zn_6.1_ (100), Pd_3.9_Zn_6.1_ (110), Pd_3.9_Zn_6.1_ (200) and Pd_3.9_Zn_6.1_ (211), respectively, indicating the formation of a new Pd–Zn intermetallic, Pd_3.9_Zn_6.1_ in Pd–Zn-ins/CNS-950. Moreover, very small diffraction peaks belonging to Pd (111), Pd(200) at 2θ = 40.1°and 46.7° appear suggesting the presence of a small amount of pure palladium phase. Further, as the temperature continues to increase to 1000 °C, the amount of pure Pd phase in Pd–Zn-ins/CNS-1000 increases sharply, as can be seen from Figure 5. The fundamental reason for this type of Pd–Zn intermetallic compound to change with carbonization temperature is that zinc is a metal that is easily vaporized, the higher the carbonization temperature is, the more zinc is vaporized. In addition, from the full width at half maximum of the main diffraction peaks, the average crystal sizes of the of the main crystalline phases for all the four samples, Pd–Zn-ins/CNS-650, Pd–Zn-ins/CNS-800, Pd–Zn-ins/CNS-950, and Pd–Zn-ins/CNS-1000 were estimated. The calculation results are 10.8 nm (PdZn in Pd–Zn-ins/CNS-650), 12.3 nm (PdZn in Pd–Zn-ins/CNS-800), 17.0 nm (Pd_3.9_Zn_6.1_ in Pd–Zn-ins/CNS-950) and 11.4 nm (Pd_3.9_Zn_6.1_ in Pd–Zn-ins/CNS-1000) using Scherrer’s equation.

**Figure 6:**
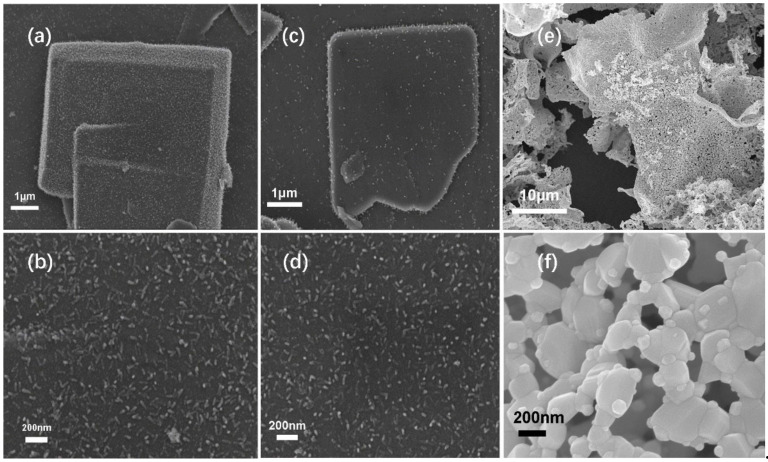
SEM images of (**a,b**) Pd–Zn-ins/CNS-650, (**c,d**) Pd–Zn-ins/CNS-850, and (**e,f**) Pd–Zn-ins/CNS-950.

SEM images of Pd–Zn-ins/CNS-650, Pd–Zn-ins/CNS-800, Pd–Zn-ins/CNS-950 are shown in Figure 6. It can be seen that the nanosheet structure remains basically intact after carbonization at 650 °C and 850 °C. A large number of nanoparticles are embedded on the surface of the carbon nanosheet in the form of nanorods. The length and the cross-sectional diameter of the rod-like nanoparticles in the Pd–Zn-ins/CNS-650 and Pd–Zn-ins/CNS-800 samples are both ~80nm and ~ 20 nm (Figure 6a–d). However, the morphology and structure of the sample, Pd–Zn-ins/CNS-950, obtained by the carbonization of Zn–TCPP(Pd) MOF nanosheets at 950 °C, have changed greatly compared with those before carbonization, and are also much different from those obtained by carbonization at 650 °C and 800 °C (Figure 6e,f). The nanosheets were bent and deformed, and have become loose and porous. Moreover, Pd–Zn alloy particles become irregular in shape, uneven in size and are much bigger than those in Pd–Zn-ins/CNS-650 and Pd–Zn-ins/CNS-800.

The four samples, Pd–Zn-ins/CNS-650, Pd–Zn-ins/CNS-850, Pd–Zn-ins/CNS-950 and Pd–Zn-ins/CNS-1000 were employed to catalyze selective hydrogenation of acetylene under simulated front-end conditions, with a gas composition of C_2_H_2_/H_2_/C_2_H_4_/N_2_ = 1:20:19:60 (volume ratio), at a space velocity of 60,000 mL g_cat_^−1^ h^−1^.

**Figure 7:**
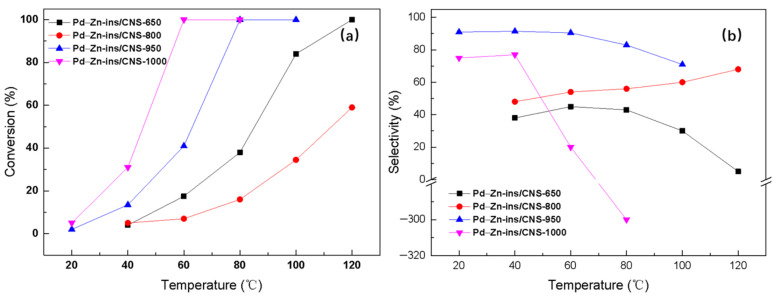
(**a**) Acetylene conversion and (**b**) ethylene selectivity over a serial of Pd–Zn-ins/CNS-X obtained at different carbonization temperatures.

As seen in Figure 7, Pd–Zn-ins/CNS-1000 shows best activity among the four samples, but its selectivity to ethylene drops dramatically when the temperature increases from 40 °C, especially at 80 °C, where it is as low as −300%. The extremely poor selectivity is attributed to the presence of large amounts of palladium nanoparticles [30,31], which has been confirmed by XRD results (Figure 5). Pd–Zn-ins/CNS-800 shows the worst catalytic activity, even when the reaction temperature reaches 120 °C, the conversion of acetylene is only 59%. Interestingly, the catalytic activity of Pd–Zn-ins/CNS-650 is better than that of Pd–Zn-ins/CNS-800, though the selectivity to ethylene over Pd–Zn-ins/CNS-650 is comparatively worse. It is suspected that during carbonization of Zn–TCPP(Pd) MOF at 800 °C, excessive Zn atoms in the system migrate to the surface of the Pd–Zn bimetallic nanoparticles due to vaporization, thus covering the palladium atoms and hindering the catalytic effect of Pd [37]. Pd–Zn-ins/CNS-950 possesses the best comprehensive catalytic performance among all the samples. Acetylene can be completely converted over Pd–Zn-ins/CNS-950 at only 80 °C, surprisingly, the corresponding selectivity is still maintained at 83%. Notably, even at 100 °C, the selectivity towards ethylene is still up to 71%, indicating a large operating window [28]. That is, even though the operating temperature fluctuates greatly, as much as a 20 °C deviation in our case, the reaction still maintains high selectivity to ethylene, and may still meet the production requirements. As far as we know, the catalytic performance of Pd–Zn-ins/CNS-950 for selective hydrogenation of acetylene under simulated front-end conditions is also much better than those of most similar catalyst systems reported in literature [30,31,33,34,35,36,37]. Combining the characterization results, Pd–Zn intermetallic, Pd_3.9_Zn_6.1_ in Pd–Zn-ins/CNS-950 should be one of the key factors leading to the excellent catalytic performance. This study provides a useful reference for the preparation of Pd–Zn intermetallics with high catalytic performance for selective hydrogenation of acetylene.

## 3. Experimental Sections

### 3.1. Chemicals

Pd(II) tetra(4-carboxyphenyl)porphin (TCPP(Pd)) (98%) and zinc nitrate hexahydrate (98%) were purchased from J&K Scientific (Beijing, China) and Shanghai Aladdin Bio-Chem Technology Co., Ltd. (Shanghai, China), respectively. The solvents applied, including methanol and N,N-dimethylformamide, were both analytical grade and supplied by Tianjin Kermel Chemical Reagent Co., Ltd. (Tianjin, China). All the chemicals were used as received without any further purification. Milli-Q water (18.2 MΩ·cm, Milli-Q System, Millipore, USA) was used in all the experiments.

### 3.2. Synthesis of Zn–TCPP(Pd) MOF Nanosheets

First, 8.97 mg of Pd-TCPP (0.01 mmol) and 11.9 mg of Zn(NO_3_)_2_·6H_2_O (0.04 mmol) were dissolved in 18 mL of the mixture of DMF and ethanol (*v:v* = 3:1) in a 20 mL Teflon vial. After stirring the mixture thoroughly, the vial was capped and placed into a stainless-steel hydrothermal cauldron followed by heating at 80 °C for 24 h. After the cauldron cooled naturally to room temperature, the resulting blood red product was washed three times with methanol, collected by centrifuging at 12,000 rpm for 5 min, and then dried at 85 °C in oven to a constant weight.

### 3.3. Preparation of Pd–Zn-ins/CNS

A total of 0.1g of the prepared Zn–TCPP(Pd) MOF nanosheets were slowly poured into a quartz tube and held in place by quartz wool. Subsequently, the tube was heated to a certain temperature at a rate of 1 °C/min, then held for 3 h. The system was protected by N_2_ with 30 mL/min throughout the process. After carbonization, the samples were denoted as Pd–Zn-ins/CNS-X, where X°C represented the final temperature of carbonation.

### 3.4. Characterization

By means of a mass spectrometry with inductively coupled plasma (iCAP RQ ICP-MS, Thermo Scientific, Waltham, MA, USA), the molar ratios of the metals Pd/Zn in the prepared 2D bimetallic MOF nanosheets were measured. Powder X-ray diffraction (XRD) patterns of the resulting samples were recorded on a Bruker D8 Advance X-ray diffractometer (Bruker AXS GmbH, Karlsruhe, Germany) with Cu Kα (λ = 1.5432 Å) radiation, operating at 40 kV and 40 mA. The patterns were collected with a scanning range (2θ) of 5°–80° and a step interval of 0.02°. Morphology of all samples was characterized by a Carl Zeiss Sigma 500 field emission scanning electron microscope (FE-SEM, Carl Zeiss AG, Oberkochen, Germany). Transmission electron microscopy (TEM) images and energy dispersive X-ray spectroscopy (EDS) elemental mappings were obtained using a Jeol JEM-2100F TEM (Jeol Ltd., Tokyo, Japan) equipped with an EDS operated at 200 KV. Fourier transform infrared (FT-IR) spectroscopy was carried out with a Nicolet 4700 FTIR spectrometer (Thermo Scientific, Waltham, MA, USA) to investigate the formation of Zn–TCPP(Pd) MOF. Adsorption and desorption isotherms of nitrogen were measured by an ASAP 2020 Plus automated apparatus (Micromeritics Instrument Corporation, Norcross, USA) at −195 °C. The specific surface area was calculated based on the Brunauer–Emmett–Teller (BET) theory model. Total pore volume (Vt) was estimated by single-point method from the amount adsorbed at P/P_0_ = 0.975. Pore size distribution was calculated using the non-local density functional theory (NLDFT) model.

### 3.5. Catalyst Evaluation

The selective hydrogenation of acetylene over Pd–Zn-ins/CNS was carried out in a fixed-bed quartz tubular microreactor (i.d. 4 mm) at atmospheric pressure. The experimental details have been described in our previous work [30], but there was a slight change here. Prior to running the reaction, 30 mg of the resulted Pd–Zn-ins/CNS was reduced in situ at 300 °C with flowing 5 vol % H_2_/N_2_ (24 mL min^−1^) for 3 h, followed by cooling to 20°C under the protection of pure N_2_ (18 mL min^−1^). Subsequently, the feed gas (30 mL/min; volume ratio of C_2_H_2_/H_2_/C_2_H_4_/N_2_ = 1:20:19:60) was introduced into the microreactor. The gas purities were as follows: N_2_ (UHP, 99.999%), H_2_ (UHP, 99.999%), C_2_H_2_ and C_2_H_4_ (mixture of 5.00 vol % C_2_H_2_ in C_2_H_4_ from Xing Rui Special Gases Co., Ltd.). The catalytic performance was tested from 30 to 120 °C. The gas components at the inlet and outlet of the reactor were analyzed by an online gas chromatography (Shimadzu GC 17A) equipped with a flame ionization detector (FID). The sampling interval temperature of the outlet components was 20 degrees. At each temperature point, the data are continuously and in parallel collected three times. A small amount of oligomers, the so called “green oil”, formed during the hydrogenation process and could be ignored according to previous product analysis [30,31,34,35]. The calculation methods for the conversion and selectivity were as follows:

(1) $$C_{2}H_{2}\;\mathrm{conversion}=\frac{C_{2}H_{2}(\mathrm{in}\;\mathrm{feed})-C_{2}H_{2}(\mathrm{in}\;\mathrm{products})}{C_{2}H_{2}\left(\mathrm{in}\;\mathrm{feed}\right)}$$

(2) $$C_{2}H_{4}\;\mathrm{selectivity}=(1-\frac{C_{2}H_{6}(\mathrm{in}\;\mathrm{products})-C_{2}H_{6}(\mathrm{in}\;\mathrm{feed})}{C_{2}H_{2}(\mathrm{in}\;\mathrm{feed})-C_{2}H_{2}(\mathrm{in}\;\mathrm{products})})$$

## 4. Conclusions

In summary, Zn–TCPP(Pd) MOF nanosheets were synthesized via a solvothermal method only using Zn(II) nitrate hexahydrate and Pd(II) tetra(4-carboxyphenyl)porphin (TCPP(Pd)) in the mixed solvent of DMF and ethanol. Through the comparison and analysis of FT-IR spectra, TEM-EDS mapping images and ICP data, the molecular structure of the prepared Zn–TCPP(Pd) product was proposed. Moreover, the crystal structure, morphology and pore structure of the MOF product were characterized in detail. The morphology of the prepared nanosheets and relatively large specific surface area (125.6 m^2^g^−1^) of the Zn–TCPP(Pd) MOF would allow it to have important individual applications. Further, XRD results show that using the prepared MOF nanosheets as the precursor, samples carbonized at different temperatures contain different types of Pd–Zn intermetallics. Among all the carbonized samples, Pd–Zn-ins/CNS-950 has the best catalytic performance of selective hydrogenation of acetylene under simulated front-end conditions. Over Pd–Zn-ins/CNS-950, acetylene can be completely converted at 80 °C with selectivity of up to 83%; even at 20 °C above the complete conversion temperature the selectivity towards ethylene can still be maintained at more than 70%, showing a large operating window [28]. This should be attributed to the existence of major phase Pd_3.9_Zn_6.1_ in Pd–Zn-ins/CNS-950_._

## Figures and Tables

**Table 1:** Molar ratios of Pd/Zn, and pore structure data of the prepared Zn–TCPP(Pd) MOF nanosheets.

Sample	Pd/Zn Molar Ratio	S_BET_ (m^2^ g^−1^)	Average Pore Size (nm)	Pore Volume (cm^3^ g^−1^)
Zn–TCPP(Pd)	~1/2	125.6	2.3	0.08

## Data Availability

Not applicable.
